# Evidence on the Need for an Integrated Approach to the Management of Diabetes: The Diagnostic Perspective if Osteomyelitis is Suspected

**Published:** 2020-02-20

**Authors:** BR Saleem

**Affiliations:** 1 Department of Surgery (Division of Vascular Surgery), University Medical Centre Groningen, University of Groningen, the Netherlands

## Background

According to the International Diabetes Federation 9.3% of adults aged 20–79 years (463 million people worldwide) are living with diabetes and the number is still raising: this will lead to an increased number of expected complications, including infections.^1^ Besides, at least half of all amputations occur in people with diabetes, most commonly because of an infected diabetic foot ulcer. Diabetic foot osteomyelitis (DFO) remain the most frequent diabetic complication, associated with substantial morbidities, requiring hospitalization, daily wound care, antimicrobial therapy and is the most common precipitating event leading to lower extremity amputation. DFO is associated with high health care costs.^2^–^3^

Diagnosing a soft tissue diabetic foot infection clinically, based on the presence of local or systemic signs and symptoms of inflammation. However, in a person with diabetes and suspected osteomyelitis of the foot the diagnosis is not easy.^4^ A combination of the probe-to-bone test (PTB), the erythrocyte sedimentation rate (or C-reactive protein), and plain X-rays as the initial studies to diagnose osteomyelitis. PTB test is the most useful, but the performing clinician’s technique and experience, the ulcer’s location and its aetiology may affect the test’s reliability. A systematic review of the PTB test found that for detecting DFO the sensitivity was 0.87 and specificity 0.83.^5^ For this reason imaging plays and important role to avoid misdiagnosis and unneeded amputations.

Conventional imaging techniques such as plain x-ray and magnetic resonance imaging (MRI) are most frequently used. If a plain x-ray, clinical and laboratory findings are mostly compatible with osteomyelitis, no further imaging is needed. The advantage of plain x-ray is that it is fully available and has a relatively low cost. The timing of the x-ray greatly influences its usefulness, as longer-standing cases are far more likely to show bony abnormalities than those present for less than 2–3 weeks. When there is doubt about the diagnosis and more imaging is needed, MRI can be considered, with a sensitivity of 0.9 and specificity of 0.8; for this reason, MRI has been the most widely used test for last decades.^6^ However, MRI positive predictive value is lower in the presence of reactive bone marrow oedema from non-infectious pathologies, such as Charcot neuroarthropathy.

More advanced techniques are available, although more expensive and difficult to interpret by non-expert radiologists. 18F-Fluoro-D-deoxyglucose positron emission tomography (18F-FDG-PET) with or without diagnostic contrast enhanced CT (18F-FDG-PET/CT), and white blood cell scintigraphy (WBCS), can be combined with single photon emission computed tomography (SPECT/CT) for better localization of the infection. However, these images are limitedly available, require special expertise and are more expensive. The sensitivity and specificity for the 18F-FDG PET/CT in diagnosing DFO recently published by Lauri et.al were: sensitivity, 89%; specificity, 92%.^7^ For WBC scan with 111In-oxine, the values were: sensitivity, 92%; specificity, 75%.^7^–^8^ For WBC scan with 99mTc-HMPAO, the values were: sensitivity, 91%; specificity, 92%. Finally, for MRI, the values were: sensitivity, 93%; specificity, 75%. If comparing these various modalities, they all had similar sensitivity, but 18F-FDG–PET and 99mTc-HMPAO–labeled WBC scintigraphy offers the highest specificity.^8^

## Conclusion

DFO is a rare but devastating complication. To avoid under-diagnosis and if x-ray is inconclusive more advanced images are available where 18F-FDG PET/CT and 99mTc-HMPAO–labeled WBC scintigraphy seems to offer higher specificity.

## References

[1] International Diabetes Federation (2019). Diabetes Atlas.

[2] Raspovic KM, Wukich DK (2014). Self-reported quality of life and diabetic foot infections. J Foot Ankle Surg.

[3] Peters EJ, Childs MR, Wunderlich RP, Harkless LB, Armstrong DG, Lavery LA (2001). Functional status of persons with diabetes-related lower-extremity amputations. Diabetes care.

[4] Meyr AJ, Seo K, Khurana JS, Choksi R, Chakraborty B Level of Agreement With a Multi-Test Approach to the Diagnosis of.

[5] Lam K, van Asten SA, Nguyen T, La Fontaine J, Lavery LA (2016). Diagnostic Accuracy of Probe to Bone to Detect Osteomyelitis in the Diabetic Foot: A Systematic Review. Clin Infect Dis.

[6] Dinh MT, Abad CL, Safdar N (2008). Diagnostic accuracy of the physical examination and imaging tests for osteomyelitis underlying diabetic foot ulcers: meta-analysis. Clin Infect Dis.

[7] Lauri C, Tamminga M, Glaudemans AWJM (2017). Detection of Osteomyelitis in the Diabetic Foot by Imaging Techniques: A Systematic Review and Meta-analysis Comparing MRI, White Blood Cell Scintigraphy, and FDG-PET. Diabetes care.

[8] Arnon-Sheleg E, Keidar Z (2018). Diabetic Foot Infection: The Role of PET/CT Imaging. Curr Pharm Des.

